# Co-Expression of IL-2 Enhances the Efficacy of FLT3-CAR-γδT Cells in Acute Myeloid Leukemia

**DOI:** 10.3390/cancers18060901

**Published:** 2026-03-11

**Authors:** Xiaona Wang, Fengtao You, Yulan Gu, Xiaofei Ma, Licui Jiang, Hai Wu, Gangli An, Xiaopeng Tian, Lin Yang

**Affiliations:** 1 Cyrus Tang Medical Institute, Collaborative Innovation Center of Hematology, State Key Laboratory of Radiation Medicine and Protection, Soochow University, Suzhou 215123, China; wxna99@163.com (X.W.); guyulan2022@163.com (Y.G.); mxfydsa@163.com (X.M.); gangli_an@suda.edu.cn (G.A.); 2 PersonGen BioTherapeutics (Suzhou) Co., Ltd., Suzhou 215123, China; fengtao.you@persongen.com (F.Y.); licui.jiang@persongen.com (L.J.); hai.wu@persongen.com (H.W.); 3 Department of Hematology, The First Affiliated Hospital of Soochow University, Jiangsu Institute of Hematology, Suzhou 215006, China; 4 National Clinical Research Center for Hematologic Diseases, Jiangsu Institute of Hematology, The First Affiliated Hospital of Soochow University, Suzhou 215006, China; 5 Institute of Blood and Marrow Transplantation, Collaborative Innovation Center of Hematology, Soochow University, Suzhou 215123, China; 6 PersonGen.Anke Cellular Therapeutics Co., Ltd., Hefei 230088, China

**Keywords:** FLT3, CAR-γδT cells, AML, IL-2, IL-7

## Abstract

**Background**: B-cell malignancies have been effectively treated using chimeric antigen receptor-T (CAR-T) treatment employing traditional αβT cells. However, because of several obstacles, application in acute myeloid leukemia (AML) is still restricted. A safer “off-the-shelf” alternative can be supplied by CAR-γδT cells, which have major histocompatibility complex (MHC)-independent tumor identification capabilities and a decreased risk of graft versus host disease (GvHD). This study aimed to develop FLT3-targeted CAR-γδT cells that co-express cytokines (IL-2 or IL-7) to increase their anti-AML persistence and therapeutic efficacy. **Methods**: FLT3-CAR-γδT cells, FLT3-IL2-CAR-γδT cells, and FLT3-IL7-CAR-γδT cells were constructed. Their antitumor potency was comprehensively assessed through cytotoxicity assays, cytokine release, and persistence evaluation in vitro (using AML cell lines and primary AML cells) and in vivo (via mouse model). **Results**: Superior cytotoxicity against AML cell lines (OCI-AML3, MOLM-13, THP-1, and MV4-11) was demonstrated by FLT3-IL2-CAR-γδT cells, which also released higher levels of granzyme B, interferon-γ (IFN-γ), and tumor necrosis factor-α (TNF-α). FLT3-IL2-CAR-γδT cells exhibited cytotoxicity in some primary AML cells in vitro. During the antigen-repeated stimulation assay, FLT3-IL2-CAR-γδT cells preserved the stem cell-like memory T (T_SCM_) cell subsets, sustained cytokine release, and maintained excellent viability. FLT3-IL2-CAR-γδT cells considerably slowed the development of AML in vivo and extended the existence (>68 days) of mice. **Conclusions**: FLT3-IL2-CAR-γδT cells exhibit potent and durable anti-AML activity, providing a novel strategy for clinical AML immunotherapy.

## Simple Summary

Adoptive cell therapy strategies based on conventional αβT cells have achieved remarkable success in other hematological malignancies, but several limiting factors have hindered their clinical application in acute myeloid leukemia. γδT cells are a non-MHC-restricted T-lymphocyte subset that have a variety of killing modes to achieve their antitumor activity. Therefore, we constructed FLT3-targeting CAR-γδT cells and optimized the CAR structure to improve the activity and persistence of CAR-γδT cells. FLT3-IL2-CAR-γδT cells show the best antitumor efficacy in vitro and in vivo, providing a new strategy for the clinical treatment of acute myeloid leukemia patients.

## 1. Introduction

Clinical symptoms such as infection, anemia, and bleeding accompany the clonal growth of myeloid blast cells in bone marrow, peripheral blood, and/or other organs in AML, the most prevalent form of adult leukemia [1,2,3]. AML is becoming more prevalent as individuals age; the median age at onset is 68 years, and the overall survival rate for those over 60 years is less than 10% after 5 years [4]. Since several CAR-T products are effective in treating B-lymphoid leukemia and lymphoma, initiatives have been made to use this therapy to treat AML. In contrast to B-lymphoblastic leukemia, CAR-T therapy encounters significant obstacles in addressing AML patients, including an immunosuppressive tumor microenvironment, antigen escape, and CAR-T quality concerns, leading to the comparatively sluggish advancement of CAR-T therapy for AML treatment [5,6,7,8].

One to five percent of peripheral blood T cells are γδT cells, an innate T-cell subset that is distinct from αβT cells [9]. γδT cells can secrete various pro-inflammatory factors and adhesion factors, thereby improving the tumor microenvironment [10,11]. Endowed with diverse tumor-targeting capabilities, γδT cells mediate tumor killing through multi-pronged recognition pathways, including NKG2D, natural cytotoxic receptors (NCRs), and γδTCR-driven MHC-independent antigen engagement [10,12]. This effectively reduces the risk of antigen escape. γδT cells recognize antigens independent of the MHC and exhibit low GvHD risk. Compared with generic CAR-T, CAR-γδT cells can be employed in allogeneic therapy without gene editing, with higher safety and applicability, which reduces potential risks due to product quality [9,10,13]. Meanwhile, several preclinical data have shown that CAR-γδT cells have powerful antitumor effects [14,15,16].

Selecting the appropriate target to prepare CAR-γδT cells is crucial for widening the therapeutic window in AML by directing cytotoxic activity against leukemic cells while preserving normal hematopoiesis and reducing off-tumor effects. At present, the more popular targets include CD33, CD123, CLL-1, FLT3 (CD135), and so on [17]. One of the most promising targets for AML therapy is FLT3, which also happens to be among the most frequently mutated genes in AML patients, with around 25–30% of AML patients having this mutation [18]. Among patients with these mutations, 20–25% of patients belong have the FLT3-ITD mutation, while 5–10% of patients have the FLT3-TKD mutation [19]. These mutations lead to an unfavorable prognosis and an elevated relapse risk in patients receiving allogeneic hematopoietic stem cell transplantation and induction chemotherapy [20]. Several preclinical studies indicate that FLT3-CAR-T cells exert potent cytotoxicity against AML tumors and markedly extend the lifespans of AML-bearing mice [19,20,21,22,23].

Cytokines are a group of signaling molecules that mediate key cellular processes, including growth, differentiation, and immune responses. Cytokine (such as IL-2, IL-7, IL-10, IL-15, and IL-18) engineering in CAR cell therapy for cancer to increase antitumor activity has been reported [24]. IL-2 is essential for the growth of effector T lymphocytes and was the first cytokine to be shown to stimulate T-cell proliferation [25,26]. Clinical data also indicate that IL-2 may enhance the antitumor effects of Vγ9Vδ2T cells without causing considerable damage by promoting their expansion in vitro and in vivo [27]. IL-7, a cytokine crucial to lymphocyte maturation as well as persistence, interacts with the IL-7-specific receptor (IL-7R) to maintain early stem cell survival, proliferation, and rearrangement of certain TCRs in the thymus, as well as both the continued existence and growth of mature T cells later in vivo [28,29]. Expansion of Vδ1T cells is reportedly promoted by IL-7 [30]. IL-10, IL-15, and IL-18 can promote the expansion of CAR-T cells to varying degrees. However, the higher the expression level of IL-10 in the patient’s plasma, the worse the prognosis of AML patients [31]. Christodoulou I et al. reported that early death occurred in mice treated with IL-15-secreting CAR-NK cells for AML [32]. Although IL-18 can enhance IFN-γ production and restore exhausted CAR-T cells, its proinflammatory effects may trigger cytokine release syndrome (CRS) [33,34]. As a result, we engineered FLT3-CAR-γδT cells that co-express IL-2 and IL-7 to improve the antitumor ability and longevity of γδT cells. Through systematic evaluations, FLT3-IL2-CAR-γδT cells emerged as the most efficacious, demonstrating enhanced cytotoxicity, cytokine release, and long-term persistence.

## 2. Materials and Methods

### 2.1. Cell Lines and Culture

The American Type Culture Collection was the source of 293T cells and the AML cell lines (MOLM-13, THP-1, OCI-AML3, and MV4-11). The First Affiliated Hospital of Soochow University provided primary AML cells. Patient characteristics are presented in Supplementary Table S1. Primary AML cells were separated from patients’ peripheral blood via the Ficoll–Paque density gradient medium (Cytiva, Marlborough, MA, USA) and cultivated in the RPMI 1640 (Hyclone, Logan, UT, USA) medium containing 15% FBS (allBio, Taichung, China) or kept in liquid nitrogen.

Peripheral blood mononuclear cells (PBMCs) from healthy donors (Milestone^®^ Biotechnologies, Shanghai, China) were revived and cultured for one day in the Opti in Vitro T cell serum-free medium without cytokines (ExCell Bio, Shanghai, China). MACS sorting columns (Miltenyi Biotec, Bergisch Gladbach, Germany) were utilized to separate TCRγ/δ cells from non-TCRγ/δ cells after PBMCs were consistently co-incubated with a Biotin-Antibody Cocktail and Anti-Biotin MicroBeads (Miltenyi Biotec). The isolated γ/δT cells were resuspended and cultured in the Opti Vitro T-cell serum-free medium. The CD3/CD28 magnetic beads (Miltenyi Biotec) were used as a human T-cell activator to stimulate γδT cells, and the day of activation was recorded as Day 0. Cells were expanded using the γδT cell expansion kit (PersonGen BioTherapeutics (Suzhou) Co., Ltd., Suzhou, China). In an incubator with 5% CO_2_, the aforementioned cells were cultivated at 37 °C. More than three healthy donors were used for γδ T cell isolation, and one donor was used for repeated experiments.

### 2.2. CAR Constructs and Transduction

**Figure 1:**
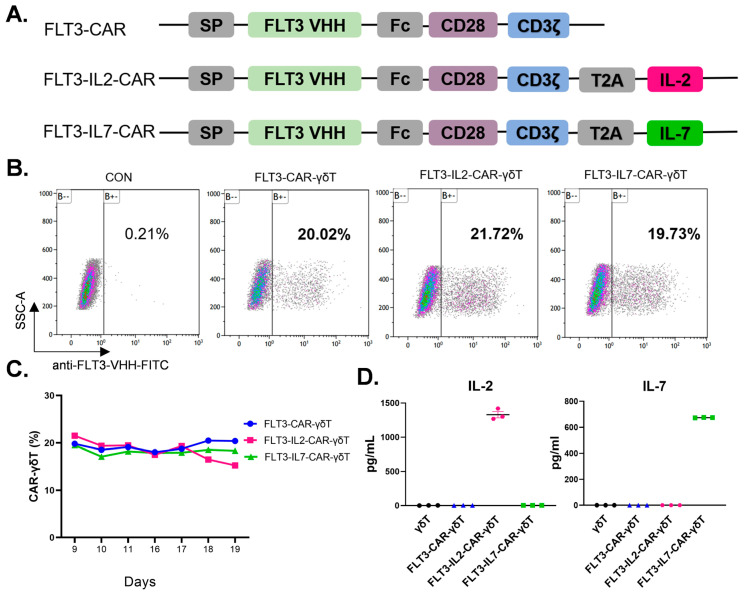
Preparation and identification of FLT3-CAR-γδT cells with different constructs. (**A**) Schematic diagram of FLT3-CAR, FLT3-IL2-CAR, and FLT3-IL7-CAR: Fc is the hinge region; CD28 is the co-stimulatory structural domain; CD3ζ is the intracellular structural domain. (**B**) Flow cytometry analysis of the positive rate of CAR-γδT cells on Day 9. (**C**) The positive rate of CAR-γδT cells at different time points during in vitro culture. (**D**) IL-2 and IL-7 concentrations in the cell culture supernatant of γδT cells and CAR-γδT cells from Days 5 to 8. The number of cells in each well is 5 × 10^5^.

The structures of FLT3-CAR, FLT3-IL2-CAR, and FLT3-IL7-CAR are shown in Figure 1A. The FLT3-CAR construct features an anti-FLT3 VHH nanobody extracellular domain linked to intracellular CD28 and CD3ζ signaling domains. The protein sequences of the cytokines IL-2 and IL-7 were added to the structure of FLT3-CAR and named FLT3-IL2-CAR and FLT3-IL7-CAR, respectively. The above sequences were designed and synthesized (GENEWIZ, Suzhou, China) and then cloned directly into lentiviral vectors. In subsequent experiments, these plasmids were used for lentivirus packaging following our group’s previous lentiviral packaging process [35].

Lentiviral concentrates were co-incubated with γδT cells for one day 48 h after γδT cell activation. Then they were washed once using PBS buffer, and the cell precipitates were resuspended in the Opti Vitro T-cell serum-free medium. Seven days after the transduction of γδT cells, CAR^+^ cells were labeled using an antibody against FLT3-VHH; then their positivity was detected using flow cytometry (Beckman, Brea, CA, USA).

### 2.3. CAR-γδT Cell Toxicity Assay In Vitro

In the killing assay, CellTrace^TM^ Violet antibody-labeled target cells were inoculated into 24-well plates at a concentration of 2 × 10^5^/well, followed by the addition of a specified number of effector cells to the respective wells at effector-to-target (E:T) ratios of 1:10, 1:5, and 1:1. The specific lysis of tumor cells was assessed using flow cytometry after a 24 h incubation period. The Annexin V/7-AAD reagent was utilized to treat the cells. Experiments were performed in three independent biological replicates, with three technical replicates each.

Primary AML cells obtained from seven patients were used in cytotoxicity assays. The expression percentages of FLT3 and CD33 in primary tumor cells are shown in Supplementary Table S1. Target cells were added to 24-well plates at a concentration of 1.5–2 × 10^5^ cells/well, depending on the quantity of the primary tumor cells. The same amount of Violet-labeled effector cells was added, and the proportion of CD33^+^ tumor cells was determined 24 h later using flow cytometry [19]. Due to the different expression levels of CD33 in the primary AML cells, it is impossible to simply compare the changes in the proportion of CD33^+^ cells. Therefore, we calculated the killing rate of the effector cells using the following formula:

Specific lysis (%) = (proportion of CD33^+^ tumor cells without effector cells added—proportion of CD33^+^ tumor cells with effector cells added) × 100/proportion of CD33^+^ tumor cells without effector cells added. Each patient sample was analyzed in three technical replicates.

In the independent experiment, the killing of the target cells was normalized.

### 2.4. CAR-γδT Cell Persistence Assay In Vitro

We created an antigen-repetitive stimulation assay to investigate the in vitro persistence of CAR-γδT cells. The CellTrace^TM^ Violet antibody was used to label the effector cells, which were added to 24-well plates at a concentration of 6 × 10^5^ cells/well. OCI-AML-3 was administered to the corresponding wells at a 1:2 E:T ratio, and five parallel sets of experiments were set up at the same time. After 24 h, OCI-AML-3 was added to four parallel sets of plates at a concentration of 6 × 10^5^ cells/well. The levels of 7-AAD and Annexin V protein expression on effector and target cells in untreated parallel plates were detected using flow cytometry. This was repeated every 24 h until five additions of OCI-AML-3 cells were completed.

### 2.5. Analysis of Cytokine Release

Culture supernatants from γδT and CAR-γδT cells (Days 5–8) were collected for assessment of IL-2 and IL-7 levels according to the CBA kit’s (BD Biosciences, San Jose, CA, USA) instructions.

Granzyme B, IFN-γ, and TNF-α cytokine levels were measured using the CBA kit after tumor-cell and effector-cell co-culture supernatants were collected. Data were analyzed using FCAP Array v3.0 software software (BD Biosciences).

### 2.6. Flow Cytometry Analysis

After being rinsed with PBS buffer, the γδT/CAR-γδT/primary AML cells were treated with the matching antibodies at 4 °C for half an hour, followed by two washes with PBS buffer. Finally, expression of the relevant proteins was assessed and quantified by flow cytometric analysis. The TCR Vδ1 antibody (Invitrogen, Carlsbad, CA, USA), the TCR-αβ antibody (BD Biosciences), the TCR Vδ2 antibody (Biolegend, San Diego, CA, USA), and the CD3 antibody (BD Biosciences) were employed to measure the purity and proportion of γδ cells. The CD45RO and CD62L antibodies (both sourced from BD Biosciences) signify the memory phenotype of effector cells. The expression level of immune checkpoint molecules was ascertained using the LAG-3 antibody (Biolegend), the TIM-3 antibody (BD Biosciences), and the PD-1 antibody (BD Biosciences). The presence of related proteins on tumor cells was monitored using the following antibodies: CD33 (BD Biosciences), Flt2/Flt3 (BD Biosciences), MICA/B (Biolegend), CD1c (BD Biosciences), CD1d (BD Biosciences), CD155 (Biolegend), and CD112 (Biolegend). The CellTrace^TM^ Violet antibody (Invitrogen) was used to distinguish between effector cells and tumor cells.

### 2.7. Animal Experiments

A number of 6–8-week-old female NPG (NOD.Cg-Prkd^CSCid^IL2rg^tm1^/Vst) mice (Beijing Vitalstar Biotechnology Co., Ltd., Beijing, China) were used to establish an AML model. NPG mice were randomly divided into 5 groups: the PBS group, the γδT cells group, the FLT3-CAR-γδT cell group, the FLT3-IL2-CAR-γδT cell group, and the FLT3-IL7-CAR-γδT cell group. On Day 0, 1 × 10^6^ OCI-AML3-luciferase cells were administered intravenously to mice, followed by the intravenous administration of 7 × 10^6^ CAR-γδT cells on Day 10. The IVIS imaging equipment (PerkinElmer, Waltham, MA, USA) was used to collect the imaging data. The long-term status and weight of mice were consistently evaluated. The percentage of γδT cells in the peripheral blood of mice at Days 7, 14, and 19 after CAR-γδT cell infusion was detected using flow cytometry. The above-mentioned experiments were conducted by investigators blinded to group allocation.

### 2.8. Statistical Analysis

All representative experiments were performed in at least three independent replicates. We utilized GraphPad Prism 8.4.0 to examine all of the statistical data in this research. The data was analyzed using one-way ANOVA or two-way ANOVA for data between multiple groups and Log-rank (Mantel–Cox) for survival data. A *p*-value of less than 0.05 was considered statistically significant.

## 3. Results

### 3.1. FLT3-CARs Expressed on γδT Cell Surface

We first constructed FLT3-CAR using FLT3 nanobody sequences (Figure 1A). IL-2 and IL-7 promote the survival and proliferation of γδT cells, thereby augmenting their antitumor efficacy in vitro [27,28,29,30]. Therefore, we added the IL-2 and IL-7 protein sequences to FLT3-CAR and named them FLT3-IL2-CAR and FLT3-IL7-CAR, respectively (Figure 1A). In addition, we detected the purity and subtype of γδT cells at the endpoint of in vitro culture and found that highly purified γδT cells with a Vδ1-type predominance could be obtained by our culture protocol (Supplementary Figure S1). Subsequently, we determined that all three CAR designs could be stably expressed in γδT cells after evaluating the transduction effectiveness of three CAR-γδT cells (Figure 1B,C). IL-2 and IL-7 levels in the supernatants of all groups were measured by CBA. The results indicated that FLT3-IL2-CAR-γδT and FLT3-IL7-CAR-γδT cells could autocrine the IL-2 and IL-7 cytokines, respectively (Figure 1D). In conclusion, we successfully constructed FLT3-CAR-γδT, FLT3-IL2-CAR-γδT, and FLT3-IL7-CAR-γδT cells.

### 3.2. FLT3-IL2-CAR-γδT Cells Eliminate AML Cell Lines In Vitro

**Figure 2:**
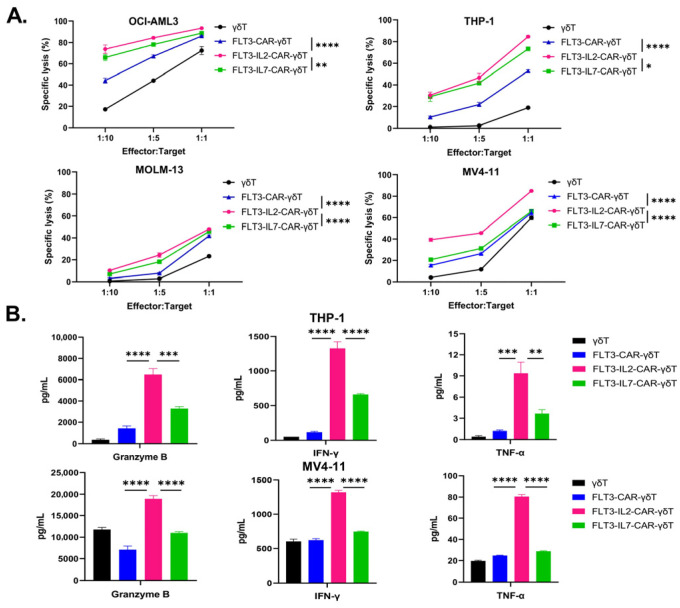
Cytotoxicity of FLT3-CAR-γδT cells with different constructs against AML cell lines in vitro. (**A**) The cytotoxicity of CAR-γδT cells against OCI-AML 3, THP-1, MOLM-13, and MV4-11 cells. *n* = 3, two-way ANOVA, * *p* < 0.05; ** *p* < 0.01; **** *p* < 0.0001. (**B**) Release of granzyme B, IFN-γ, and TNF-α after incubation of CAR-γδT cells with THP-1 and MV4-11 cells for 24 h at an E: T ratio of 1:1. *n* = 3, One-way ANOVA ** *p* < 0.01; *** *p* < 0.001; **** *p* < 0.0001. Representative results from three independent experiments are shown.

Flow cytometry analysis revealed that all four AML cell lines highly expressed the FLT3 antigen (Supplementary Figure S2). To investigate the cytotoxicity of several FLT3-CAR-γδT cells, we incubated OCI-AML-3, THP-1, MOLM-13, and MV4-11 tumor cells with three CAR-γδT cells. The findings indicated that FLT3-IL2-CAR-γδT cells exhibited superior tumor-killing effects at E:T ratios of 1:10, 1:5, and 1:1. Specifically, at an E: T ratio of 1:1, FLT3-IL2-CAR-γδT cells could almost completely eliminate OCI-AML3, THP-1 and MV4-11 tumor cells (Figure 2A). In addition, we also examined the cytokine released in the supernatant and found that FLT3-IL2-CAR-γδT cells showed the strongest release of granzyme B, IFN-γ and TNF-α (Figure 2B). In conclusion, FLT3-IL2-CAR-γδT cells exhibited the most superior cytotoxicity against multiple AML cell lines in vitro.

### 3.3. FLT3-IL2-CAR-γδT Cells Exhibit Cytotoxicity for some Primary AML Cells In Vitro

**Figure 3:**
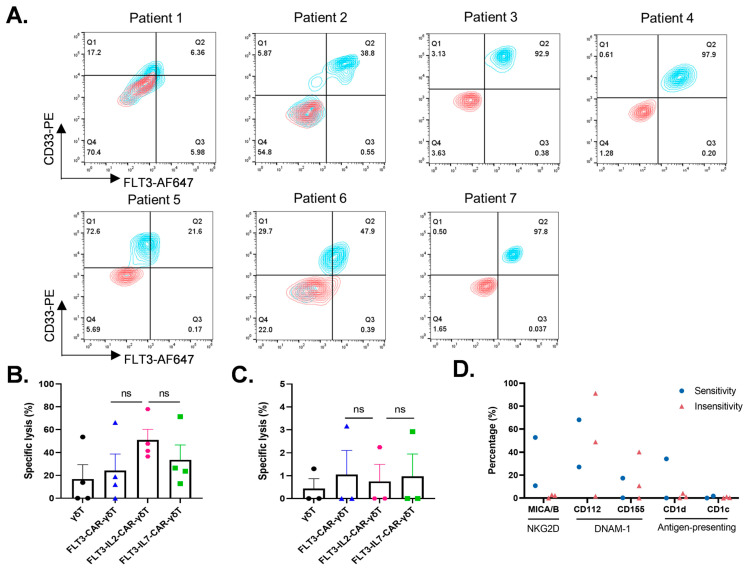
Cytotoxicity of FLT3-CAR-γδT cells with different constructs against primary AML cells in vitro. (**A**) The expression of FLT3 and CD33 in primary tumor cells. Red indicates the unstained control group, and blue indicates the experimental group after the corresponding flow antibody staining. (**B**,**C**) The killing efficiency of CAR-γδT cells against primary tumor cells. *n* = 3, One-way ANOVA, ns: not significant. (**D**) The expressions of MICA/B, CD1c, CD1d, CD112, and CD155 in primary tumor cells. MICA/B is the ligand of NKG2D. CD112 and CD155 are the ligands of DNAM-1. CD1c and CD1d are γδT cell-surface antigen-presenting proteins. The above data are presented as the mean ± SEM.

We obtained peripheral blood samples from seven AML patients to investigate the killing effect of different kinds of FLT3-CAR-γδT cells on primary AML cells in vitro (Supplementary Table S1). The surface expression of the FLT3 and CD33 antigens in each sample was assessed via flow cytometry (Figure 3A). The same number of CAR-γδT cells was incubated with primary leukemia cells, and lysis specificity was assessed by comparing the proportion of CD33^+^ tumor cells before and after killing. We found that tumor cells from four patients (Patients 1, 2, 3, and 4) were more sensitive to CAR-γδT cell killing. The killing statistics indicated that FLT3-IL2-CAR-γδT cells showed numerically higher killing activity against primary AML cells compared to other groups, though this difference was not significant. (Figure 3B). We also found that tumor cells from three patients (Patients 5, 6, and 7) were insensitive to CAR-γδT cell killing (Figure 3C). In conclusion, the results indicated that FLT3-IL2-CAR-γδT cells could exhibit cytotoxic effects on some primary AML cells, which suggests that our FLT3-IL2-CAR-γδT cells have the potential to treat AML in the clinic.

### 3.4. FLT3-IL2-CAR-γδT Cells Exhibit Significant Persistent Killing Ability In Vitro

**Figure 4:**
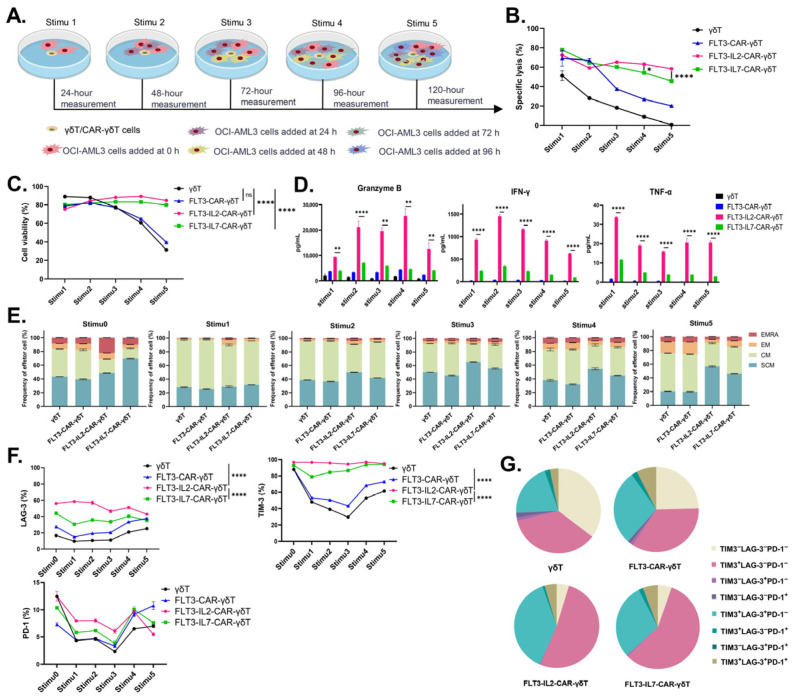
In vitro anti-tumor persistence of FLT3-CAR-γδT cells with different structures. (**A**) Schematic diagram of the antigen-repetitive stimulation assay. (**B**) Killing effect of CAR-γδT cells during repeated stimulation with tumor antigens. *n* = 3, two-way ANOVA, * *p* < 0.05; **** *p* < 0.0001. (**C**) The percentage of Annexin-V and 7-AAD double-negative cells in effector cells was detected by flow cytometry 24 h after each added tumor antigen stimulation. *n* = 3, two-way ANOVA, **** *p* < 0.0001; ns: not significant. (**D**) The release of granzyme B, IFN-γ, and TNF-α in each tumor antigen stimulation supernatant. Two-way ANOVA, ** *p* < 0.01; **** *p* < 0.0001. (**E**) Memory phenotype of effector cells after 24 h of stimulation with each tumor antigen. CD45RO^−^CD62L^+^ stem cell-like memory T cells (T_SCM_); CD45RO^+^CD62L^+^ central memory T cells (T_CM_); CD45RO^+^CD62L^−^ effector memory T cells (T_EM_); CD45RO^−^CD62L^−^ terminally differentiated T cells (T_EMRA_). (**F**) LAG-3, TIM-3, and PD-1 expression in effector cells before and 24 h after each addition of tumor antigen stimulation. *n* = 3, two-way ANOVA, **** *p* < 0.0001. (**G**) LAG-3, TIM-3, and PD-1 expression in γδT and CAR-γδT cells 24 h after the last tumor antigen stimulation. The above data are presented as the mean ± SEM. Representative results from three independent experiments are shown.

Further investigations were required to characterize the antitumor persistence of FLT3-CAR-γδT cells with different structures. We designed an antigen-repetitive stimulation assay to mimic the environment of long-term tumor stimulation in vivo (Figure 4A). The results showed that FLT3-IL2-CAR-γδT cells continued to have high killing efficiency and maintained relatively high viability during the antigen-repetitive stimulation assay (Figure 4B,C). Compared with other groups, FLT3-IL2-CAR-γδT cells could continuously release IFN-γ, granzyme B, and TNF-α at a high level (Figure 4D). These results suggested that the stable release of high-level cytokines is one of the reasons for the maintenance of the high efficiency and sustained killing ability of FLT3-IL2-CAR-γδT cells.

T_SCM_ cells are more appropriate for immunotherapy due to their significant self-renewal and proliferation capabilities, which enable them to promptly replenish a greater number of memory and effector T-cell subsets [36,37,38,39]. Therefore, we detected memory differentiation markers in γδT, FLT3-CAR-γδT, FLT3-IL2-CAR-γδT, and FLT3-IL7-CAR-γδT cells after each tumor antigen stimulation. In contrast to other groups, FLT3-IL2-CAR-γδT cells remained at a high level in the T_SCM_ cell subsets, which did not decrease after multiple tumor antigen stimulations (Figure 4E).

The expression of LAG-3 and TIM-3 in γδT, FLT3-CAR-γδT, and FLT3-IL7-CAR-γδT cells decreased after initial tumor antigen stimulation and then increased with the increase in tumor antigen stimulation times. In contrast, FLT3-IL2-CAR-γδT cells maintained high expression of LAG-3 and TIM-3 during tumor antigen stimulation (Figure 4F). . The results showed that FLT3-IL2-CAR-γδT cells had the highest percentage of cells expressing two or more immune checkpoint molecules (Figure 4G). Although PD-1, TIM-3, and LAG-3 are typical markers of αβT cell exhaustion, their roles and functions in γδT cells may differ from those in αβT cells. It has been reported that these molecules do not represent γδT cell exhaustion but may be beneficial to the antitumor activity of γδT cells [10,40].

### 3.5. FLT3-IL2-CAR-γδT Cells Have Significant Antitumor Effects In Vivo

**Figure 5:**
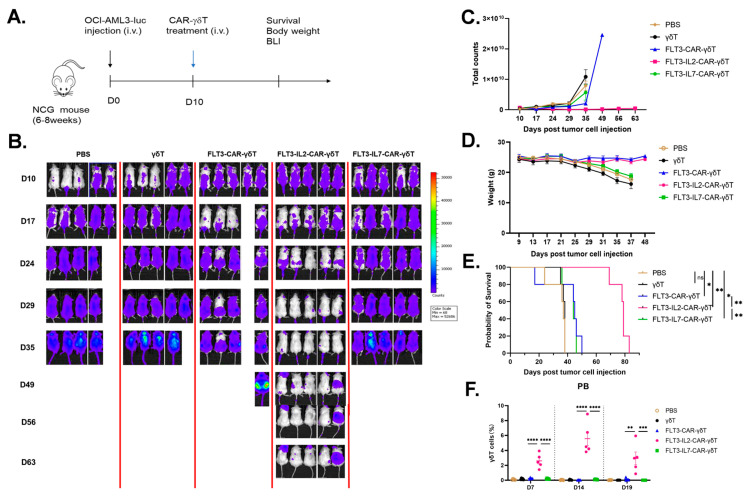
Antitumor effects of FLT3-CAR-γδT cells with different constructs in the AML model. (**A**) In vivo efficacy validation protocol. = 5 mice/group. (**B**) Bioluminescence images of mice at specified time points after tumor development. (**C**) Quantification of tumor burden in each group of mice by bioluminescence imaging. (**D**) Daily monitoring and counting of mouse body weights. (**E**) Mouse survival curves. Log-rank (Mantel–Cox), * *p* < 0.05; ** *p* < 0.01; ns: not significant. (**F**) The percentage of total γδT cells in the peripheral blood of mice on Days 7, 14, and 19 after CAR-γδT cell transfusion. One-way ANOVA, ** *p* < 0.01, *** *p* < 0.001; **** *p* < 0.0001. The above data are presented as the mean ± SEM.

To assess the in vivo efficacy of FLT3-CAR-γδT cells with varying structures, we developed a cell-derived xenograft (CDX) mouse model of AML (Figure 5A). In vivo imaging confirmed the superior tumor-suppressive efficacy of FLT3-IL2-CAR-γδT cells over other CAR-γδT cells (Figure 5B,C), together with no noticeable decrease in mouse weight (Figure 5D). The CAR-γδT groups effectively prolonged the lifespan of mice compared with the PBS and γδT groups. The FLT3-IL2-CAR-γδT group was the most effective, which remarkably extended the survival of mice to more than 68 days (Figure 5E). Meanwhile, we detected the total γδT cells in the peripheral blood of mice on Days 7, 14, and 19 after CAR-γδT cell transfusion. Flow cytometry data indicated that after 19 days of infusing CAR-γδT cells, only the FLT3-IL2-CAR-γδT group could detect γδT cells in the peripheral blood (Figure 5F). These data demonstrate that IL-2 supports the prolonged survival and expansion capacity of FLT3-IL2-CAR-γδT cells in mice. In conclusion, FLT3-IL2-CAR-γδT cells markedly suppressed tumor proliferation and extended survival in mice in vivo.

## 4. Discussion

Following the landmark success of CD19-targeting CAR-T cells, the CAR-T therapeutic approach has been actively explored for a range of malignancies, including AML. Nevertheless, CAR-T therapy encounters numerous obstacles in AML, contrasting with the significant advancements it has made in lymphoid leukemia. Specific surface antigens are lacking, and the high heterogeneity of AML antigens restricts CAR-T cell treatment in AML. Furthermore, tumor immune escape resulting from CAR-T cell therapy is also a major obstacle in AML [17]. This is also reflected in the immunosuppressive tumor microenvironment. The malignant bone marrow microenvironment in AML not only promotes the immune escape of drug-resistant leukemic cells but also drives their migration and expansion [8]. Moreover, the tumor cells in AML patients can release cytokines that impede T-cell proliferation, thereby diminishing CAR-T cell efficacy [41]. CAR-γδT cells, as a potential alternative, have garnered significant interest in the last several years. In addition to targeting tumors via CAR, CAR-γδT cells retain the capacity to recognize malignant cells through their endogenous γδT cell receptors. They can trigger several tumor-killing pathways; diversified pro-inflammatory factors and chemokines can also be secreted by activated γδT cells to establish an inflammatory environment. These advantages of CAR-γδT cells can effectively reduce tumor immune escape and improve the tumor microenvironment. Furthermore, γδT cells are not MHC-restricted and have a low risk of GvHD development. These characteristics greatly augment the safety and applicability of CAR-γδT cells in comparison to CAR-T cells and reduce the quality issues of the product. FLT3 is an AML-specific antigen, with minimal or absent expression in healthy tissues but high expression in the majority of AML tumor cells [42]. Based on this, we chose FLT3 as the target to construct CAR-γδT cells and conduct preclinical efficacy studies in AML. IL-2 and IL-7 can accelerate the expansion of γδT cells and enhance their antitumor effects [27,30]. Therefore, to further augment the activity and durability of CAR-γδT cells, we co-expressed IL-2 or IL-7 in the CAR construct to enable FLT3-CAR-γδT cells to release IL-2 or IL-7. This study mainly compared the antitumor capability of FLT3-CAR-γδT, FLT3-IL2-CAR-γδT, and FLT3-IL7-CAR-γδT cells in vitro and in vivo and screened the most effective CAR-γδT cells against AML tumor cells.

We successfully constructed FLT3-CAR-γδT, FLT3-IL2-CAR-γδT, and FLT3-IL7-CAR-γδT cells and verified the stability of CAR expression for the first time. At present, FDA-approved FLT3 inhibitors only have a significant therapeutic effect on patients with FLT3 mutations, and patients are prone to relapse after treatment, which is far from meeting the clinical needs [43,44,45]. However, these FLT3-targeting CAR-γδT cells we constructed showed high killing efficiency against both FLT3-mutant and wild-type AML tumor cell lines, accompanied by a great release of IFN-γ, granzyme B, and TNF-α. This suggests that CAR-γδT cells targeting FLT3 have a broader application prospect than FLT3 inhibitors.

In this study, we found that the killing effect of CAR-γδT cells on primary AML tumor cells may be not related to the expression level of FLT3 on the surface of the tumor cells (Figure 3B,C and Supplementary Table S1). CAR-γδT cells recognize and kill tumors not only based on the CAR structure but also based on the self-expressed receptors, such as NKG2D and DNAM-1. Subsequently, surface levels of γδT cell-targeting ligands in primary AML cells were assessed by flow cytometric analysis. Owing to insufficient cells from the primary samples, however, the expression of relevant ligands on tumor cells was assessed in only five patients. The tumor cells from two patients who were sensitive to CAR-γδT cells expressed MICA/B and CD112, and one of them also expressed CD1d and CD155. The tumor cells from two patients who were not sensitive to CAR-γδT cells expressed CD112 and CD155 but hardly MICA/B, and the other case did not express ligands associated with γδT cells. All primary AML cells did not express CD1c (Figure 3D and Supplementary Figure S3). In parallel, we found that the higher the expression of MICA/B, the better the killing effect of CAR γδT (the killing rate of FLT3-CAR-IL2-γδT cells against Patient 1’s tumor cells was 37%, and the killing rate of FLT3-CAR-IL2-γδT cells against Patient 3’s tumor cells was 48%; Supplementary Figure S3). The data imply that primary AML cells expressing MICA/B could be more susceptible to the killing effect of CAR-γδT cells. Therefore, we hypothesized that FLT3-IL2-CAR-γδT cells would be more advantageous for killing primary AML cells with high expression of the NKG2D ligand compared to those with high expression of the DNAM-1 ligand; however, whether the expression of the NKG2D ligand in primary tumor samples would directly affect the killing efficacy of FLT3-IL2-CAR-γδT cells would need to be confirmed by the results of more subsequent studies. In summary, to better clarify how FLT3-CAR-γδT cells mediate cytotoxicity against primary AML cells, future studies will need to use FLT3-knockdown cell lines to determine whether their killing activity relies on FLT3 expression, and as many primary AML samples as possible should be included. We will also perform NKG2D blockade assays to evaluate the contribution of the NKG2D pathway, especially when CAR-γδT cells target AML cells with low FLT3 expression.

In the antigen-repetitive stimulation assay, we found that FLT3-IL2-CAR-γδT cells had satisfactory effects in terms of killing persistence. FLT3-IL2-CAR-γδT cells could maintain high cell viability and were also able to stably and consistently release high levels of functional cytokines, which enhanced their killing persistence. T_SCM_ cells possess self-renewal capabilities, exhibit robust proliferation, and might timely replenish both memory and effector T cells [46]. Following multiple tumor antigen stimulations, the T_SCM_ cell subsets decreased in all groups except for FLT3-IL2-CAR-γδT cells. However, FLT3-IL2-CAR-γδT cells have maintained relatively stable T_SCM_ cell subsets, which may be one of the reasons for their maintenance of killing. T-cell depletion is most typically characterized by elevated expression of inhibitory receptors (e.g., PD-1, LAG-3, and TIM-3), and T-cell depletion in tumors negatively correlates with the treatment efficacy of the immunotherapy [47,48]. However, unlike conventional αβT cells, elevated expression of conventional inhibitory receptors in γδT cells does not necessarily indicate γδT cell depletion. De Vries N L et al. showed that PD-1^+^γδT (Vδ1/3) cells isolated from tumor tissue had a higher antitumor ability than PD-1^−^γδT (Vδ2) cells [49]. Rancan et al. showed that tumor-infiltrating PD-1^+^TIGIT^+^TIM-3^+^ Vδ2T cells have significant effector functions and highly express proliferation-related genes [40]. Similar to these findings, our results also revealed that FLT3-IL2-CAR-γδT cells with the best in vivo antitumor efficacy during antigen-repetitive stimulation also consistently expressed LAG-3 and TIM-3 at high levels. These results imply that the FLT3-IL2-CAR-γδT cell population, even with elevated LAG-3 and TIM-3 expression, remains in a functionally competent state.

In mouse experiments, we found that FLT3-IL2-CAR-γδT cells markedly suppressed tumor cell expansion and significantly extended the survival of mice with AML. In addition, γδT cells were still detected in the peripheral blood of mice 19 days after infusion of CAR-γδT cells. Preliminary RNA-seq analysis also suggested that co-expression of IL-2 upregulated genes associated with cell proliferation in FLT3-IL2-CAR-γδT cells, which is consistent with the in vivo results. It is noteworthy that FLT3-IL2-CAR-γδT cells have higher levels of IL-2 release compared to IL-7 release from FLT3-IL7-CAR-γδT cells. However, it is not yet known whether the functional effects of the same dose of IL-7 or IL-2 alone on γδT cells are similar to the results explored in this study. However, for the FLT3-CAR structure constructed in this study, FLT3-IL2-CAR-γδT cells had higher co-expressed cytokine release and more significant antitumor effects in vitro and in vivo.

Target selection has been a major factor plaguing AML therapy, and targeting normal bone marrow progenitor cells by CAR-T cells can cause hematopoietic toxicity [17]. FLT3 is highly expressed in AML tumor cells, but it is also expressed in some hematopoietic stem cells and myeloid progenitor cells [45,50]. Therefore, careful safety assessment is required prior to its clinical application. While clinical safety data for FLT3-CAR-T cells remain unavailable, preclinical evaluations demonstrate that they have only a modest impact on normal hematopoietic stem/progenitor cell populations, supporting clinical development [50]. In addition, several reports confirm that FLT3-CAR-T cells exhibit no detectable cytotoxicity against hematopoietic progenitor cells [20,22]. In addition, γδT cells are considered ideal for developing universal therapies due to their non-MHC restriction [51]. Clinical studies have demonstrated that immunotherapy based on allogeneic γδT cells has good safety and tolerability in treating B-cell lymphoma, lung cancer, hepatocellular carcinoma, and pancreatic cancer [52,53,54]. Therefore, the targeted FLT3-CAR-γδT cells we have developed may be safer and more applicable than FLT3-CAR-T cells.

Karbowski et al. used autologous T cells to generate a second-generation CAR-T cell (the scFv fragment of FLT3, the CD28 costimulatory domain, and the CD3ζ activation domain), referred to as AMG553 [50]. After 40 h of co-incubation at an E:T ratio of 1:1, AMG533 induced a cytotoxicity rate with 36–57% efficacy against the tumor cell lines MOLM-13 and MV4-11 [50]. In the present study, following 24 h of co-incubation at an E:T ratio of 1:1, FLT3-IL2-CAR-γδT cells mediated specific lysis of 48–84% of cells in the MOLM-13 and MV4-11 leukemic cell lines (Figure 2A). However, following a recent evaluation of the cell therapy strategy, the trial’s responsible party discontinued this clinical trial. Meanwhile, three clinical trials of FLT3-CAR-T cell therapy (NCT05445011, NCT06786533, and NCT06760260) are recruiting participants, but unfortunately, no data from in vitro and in vivo experiments have been reported yet. Notably, FLT3-CAR-T cells generated using the FLT3-CAR structure characterized in this study have entered clinical trials for AML therapy (NCT05445011). This CAR strategy is based on a nanobody sequence that facilitates more efficient transduction into γδT cells and confers lower immunogenicity. Although IL-2 immunotherapy has been used clinically for more than 30 years, it has only provided therapeutic benefit in some patients with melanoma and renal cell carcinoma [55]. Nevertheless, high-dose regimens pose risks, including capillary leak syndrome [56,57]. However, preclinical data have demonstrated that CAR-Vδ1 T cells co-expressing IL-2 exhibit a favorable safety profile in solid tumor therapies [58]. However, the safety of FLT3-IL2-CAR-γδT cells should be carefully evaluated before clinical application.

## 5. Conclusions

In conclusion, we constructed three types of FLT3-targeting CAR-γδT cells. Among these, FLT3-IL2-CAR-γδT cells represent a promising “off-the-shelf” therapeutic option for AML by combining potent antitumor activity, durability, and reduced toxicity. This strategy is expected to address critical challenges in AML immunotherapy and warrants further clinical investigation.

## Data Availability

Experimental data are available from the corresponding author upon reasonable request.

## Supplementary Materials

The following supporting information can be downloaded at: https://www.mdpi.com/article/10.3390/cancers18060901/s1, Figure S1: γδT cell purity and subtype assays. Flow cytometry was used to determine the purity and subtype of γδT cells cultured on Day 19 in vitro. CD3^+^ TCRαβ^−^ cells were γδT cells; CD3^+^ TCR Vδ1^+^ cells were Vδ1 T cells. CD3^+^ TCR Vδ2^+^ cells were Vδ2 T cells. CD3^+^ TCR Vδ1^−^ TCR Vδ2^−^ cells were other types of γδT cells. Figure S2: Expression of the FLT3 antigen on the surface of AML tumor cell lines. (A–D) The expression of the FLT3 antigen on the surface of the OCI-AML3 (FLT3mut^−^) cell line, the THP-1 (FLT3mut^−^) cell line, the MOLM-13 (FLT3mut^+^) cell line, and the MV4-11 (FLT3mut^+^) cell line. Figure S3: Expression of ligands associated with γδT cells on the surface of primary samples. Flow cytometry was used to detect the expression of MICA/B, CD1c, CD1d, CD112, and CD155 on the surface of primary tumor samples. Red indicates the unstained control group, and blue indicates the experimental group after staining with the corresponding flow antibody. Table S1: Clinical characteristics of patients with acute myeloid leukemia (AML).
