# Supplementary material for: Co-Expression of IL-2 Enhances the Efficacy of FLT3-CAR-γδT Cells in Acute Myeloid Leukemia
Source: Cancers (Basel). 2026 Mar 11;18(6):901. doi: 10.3390/cancers18060901 (PMC13024764; doi:10.3390/cancers18060901)
Supplement: Supplementary file 1 [file cancers-18-00901-s001.zip › cancers-4162363-supplementary.pdf]

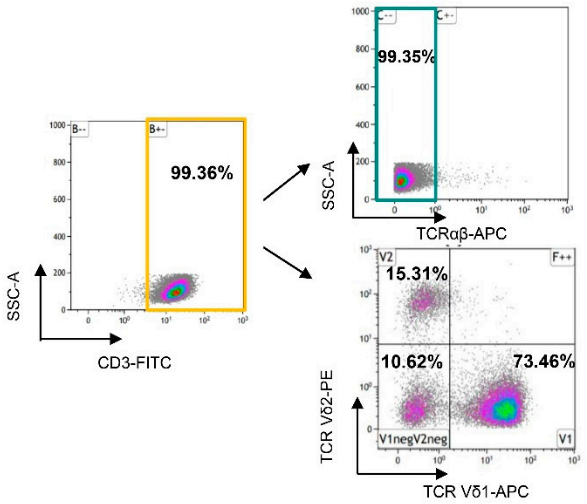

Figure S1: γδT cell purity and subtype assays. Flow cytometry was used to determine the purity and subtype of γδT cells cultured on Day 19 in vitro. $CD3^+$ $TCR\alpha\beta^-$ were γδT cells; $CD3^+$ TCR $V\delta1^+$ were Vδ1 T cells. $CD3^+$ TCR $V\delta2^+$ were Vδ2 T cells. $CD3^+$ TCR $V\delta1^-$ TCR $V\delta2^-$ were other types of γδT cells.

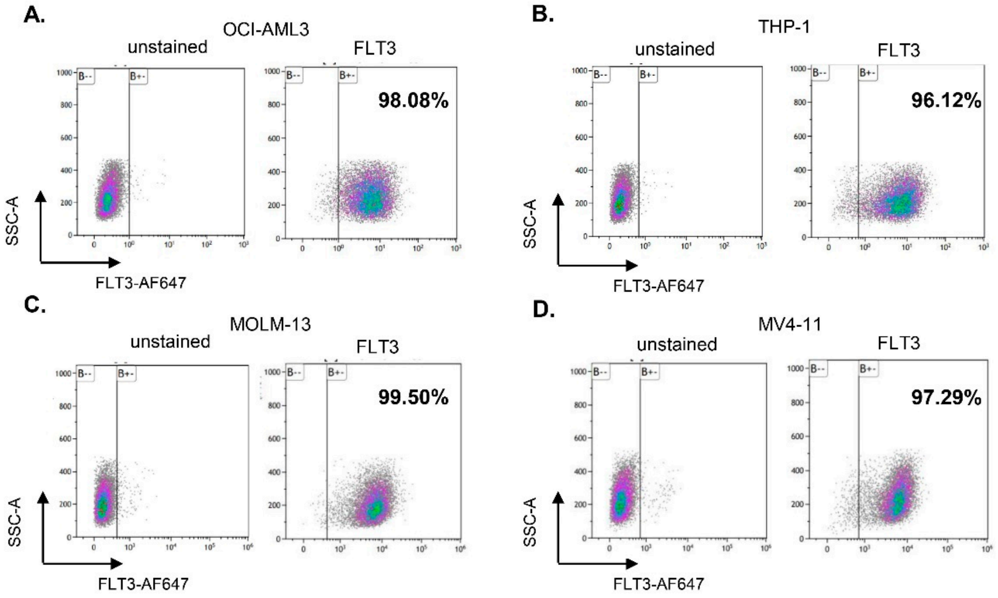

Figure S2: Expression of FLT3 antigen on the surface of AML tumor cell lines. (A-D) The expression of FLT3 antigen on the surface of OCI-AML3 ($FLT3mut^{-}$) cell line, THP-1 ($FLT3mut^{-}$) cell line, MOLM-13 ($FLT3mut^{+}$) cell line, and MV4-11 ($FLT3mut^{+}$) cell line.

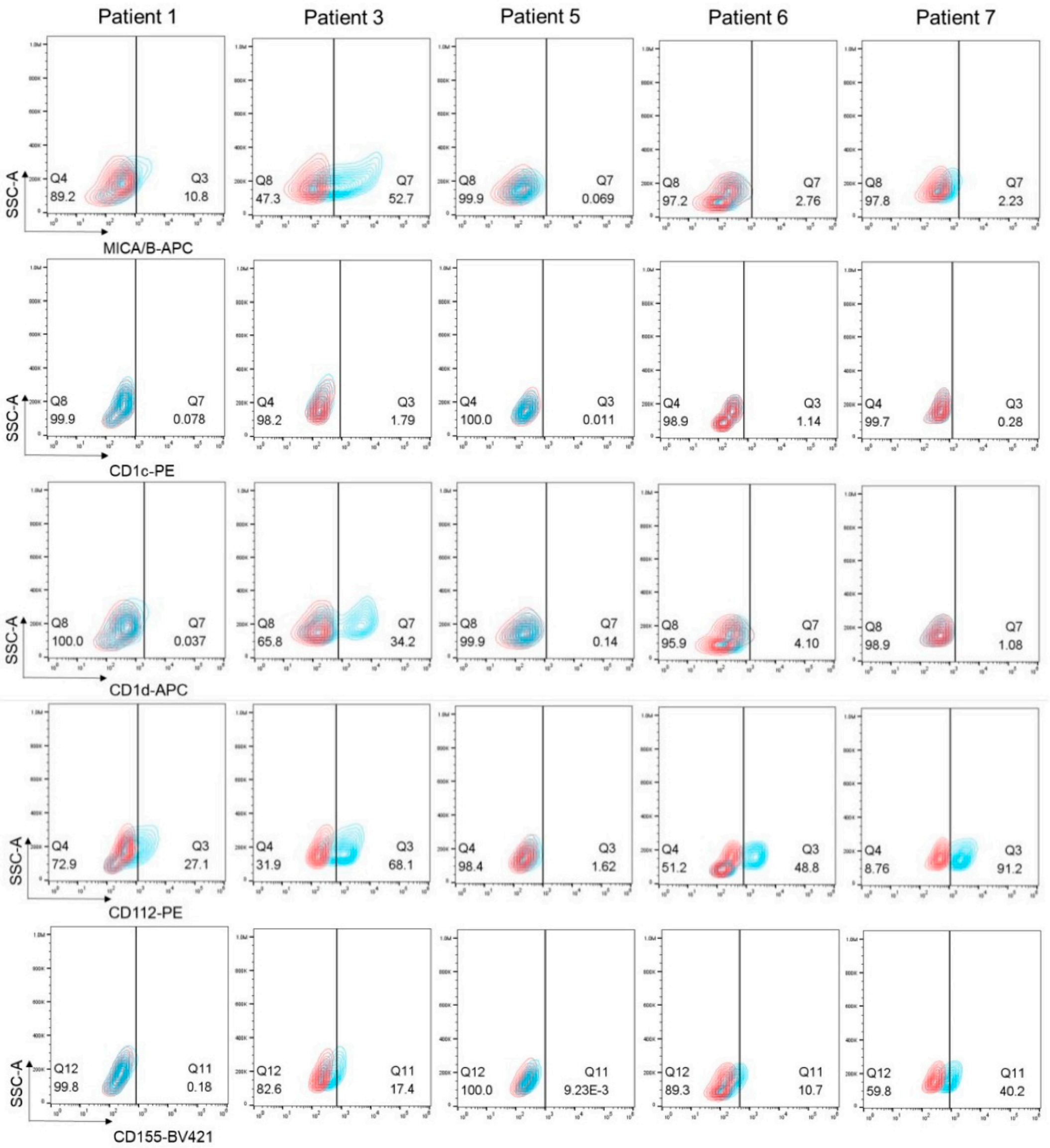

Figure S3: Expression of ligands associated with γδT cells on the surface of primary samples. Flow cytometry was used to detect the expression of MICA/B, CD1c, CD1d, CD112, and CD155 on the surface of primary tumor samples. Red indicates the unstained control group, and blue indicates the experimental group after staining with the corresponding flow antibody.

Table S1: Clinical characteristics of patients with acute myeloid leukaemia (AML)

**Patient**	**Sex**	**Age**	**FLT3$^+$ cell (%)**	**CD33$^+$ cell (%)**
1	Female	36	12.34	23.56
2	Female	45	39.35	44.67
3	Male	60	93.28	96.03
4	Male	71	98.10	98.51
5	Male	57	21.77	94.20
6	Male	14	48.29	77.60
7	Male	36	97.84	98.30
